# Clinical Usefulness of Transjugular Liver Biopsy in Patients With Hematological Diseases With Liver Dysfunction

**DOI:** 10.7759/cureus.19555

**Published:** 2021-11-14

**Authors:** Toru Ishikawa, Erina Kodama, Takamasa Kobayashi, Motoi Azumi, Yujiro Nozawa, Akito Iwanaga, Tomoe Sano, Terasu Honma

**Affiliations:** 1 Department of Gastroenterology and Hepatology, Saiseikai Niigata Hospital, Niigata, JPN; 2 Gastroenterology and Hepatology, Saiseikai Niigata Hospital, Niigata, JPN

**Keywords:** coagulation disorders, pancytopenia, transjugular liver biopsy, hematology, liver disease

## Abstract

Introduction

Transjugular liver biopsy (TJLB) is indicated for patients in whom percutaneous liver biopsy is contraindicated, such as those with hematological diseases complicated by liver dysfunction. However, the clinical utility of TJLB in this group of patients has not been thoroughly investigated. The objective of this study is to evaluate the clinical efficacy of TJLB in patients with hematological diseases complicated by liver dysfunction.

Methods

We analyzed the data of patients who developed liver disorders during treatment for hematological diseases at our hospital and required tissue diagnosis via TJLB. The clinical features of patients were analyzed.

Results

Twenty-seven patients (mean age, 60.07 years; 12 men, 15 women) requiring tissue diagnoses via TJLB after developing liver disorders while undergoing treatment for hematological diseases were enrolled. One patient with autoimmune hemolytic anemia was diagnosed with drug-induced liver injury; two patients with amyloidosis had nonalcoholic steatohepatitis; one patient with acute promyelocytic leukemia had a drug-induced liver injury; one patient with chronic myelomonocytic leukemia had liver infiltration caused by an underlying disease; three patients with idiopathic thrombocytopenic purpura had autoimmune hepatitis; four patients with malignant lymphoma had liver infiltration by the underlying disease, and one patient with multiple myeloma had liver disorder caused by disseminated intravascular coagulation. Moreover, one patient had hepatitis B reactivation, another had hepatitis E, and six patients had a drug-induced liver injury. The treatment regimen was altered in cases of liver infiltration caused by the underlying disease, and the drug was changed for patients with drug-induced liver injury.

Conclusion

The etiology of liver disorders in patients with hematological diseases varies widely. Therefore, histological diagnosis using TJLB is useful to determine an appropriate therapeutic strategy for underlying hematological diseases.

## Introduction

Liver biopsies are important for the definitive diagnosis of acute and chronic liver diseases and are generally performed percutaneously [1]. Histological evaluation of liver biopsy specimens allows clinicians to evaluate disease etiology, ascertain the degree of inflammation and fibrosis, and identify the response to treatment. Nonetheless, in patients with idiopathic liver disease, there is the risk of complications such as unexpected bleeding. Additionally, in patients at an increased risk of bleeding, such as those with ascites or advanced coagulation disorders, percutaneous liver biopsy (PLB) is generally contraindicated.

Transjugular liver biopsies (TJLBs) can be performed in patients with contraindications for PLBs such as coagulation disorder, ascites, acute liver failure, or liver transplantation [2-4]. In patients with hematological diseases and liver dysfunction, PLB is also associated with a risk of unexpected bleeding [3,4]. However, no studies have investigated the usefulness of TJLBs in patients with hematological diseases who developed severe liver dysfunction. Hence, we conducted this study with an aim of evaluating the clinical efficacy of TJLB in patients with hematological diseases who developed liver disorders.

## Materials and methods

This study was conducted in accordance with the principles of the Declaration of Helsinki, and the study protocol was approved by the ethics committee of Saiseikai Niigata Hospital (approval number: E05-13). All patients provided written informed consent prior to study participation.

We analyzed the data of 27 patients (12 men and 15 women) who developed liver disorders during treatment for hematological diseases at our hospital and who required tissue diagnosis via TJLB. The inclusion criterion was the requirement for an alternative liver biopsy procedure due to contraindications for PLBs or bleeding risk. TJLB was considered the first choice for patients in whom the administration of antithrombotic drugs could not be interrupted during the biopsy procedure regardless of the drug or dose and patients with severe accumulations of ascites. Moreover, in this study, TJLB was indicated in patients with coagulation disorders and/or thrombocytopenia of hematological disorders. The mean patient age was 60.07 (22-87) years. The underlying causative diseases were autoimmune hemolytic anemia (AIHA) in one patient, acute lymphocytic leukemia (ALL) in one patient, suspected amyloidosis in seven patients, acute promyelocytic leukemia (APL) in one patient, chronic myelomonocytic leukemia (CMMoL) in one patient, idiopathic thrombocytopenic purpura (ITP) in three patients, malignant lymphoma in 12 patients, and multiple myeloma in one patient.

TJLB was indicated for patients with diffuse liver diseases who required a biopsy and either had a contraindication to PLBs or required hemodynamic evaluation as part of their diagnostic workup. One of the most common indications for TJLB is a coagulation disorder [5]. The degree of coagulopathy at which PLBs are contraindicated varies between institutions; generally, it is advisable not to perform PLB if the international normalized ratio is > 1.5 or the platelet count is < 50,000 ´109/L [6]. Other common indications include the presence of ascites, peliosis hepatis, or morbid obesity; history of liver transplant; past PLB failure; or the need to undergo a transjugular intrahepatic portosystemic shunt procedure.

TJLB was performed by experienced hepatologists using the LABS-100 system (liver access and biopsy kit, 18 G; Cook Medical, Bloomington, IN, USA) according to the standard technique under ultrasound and fluoroscopic guidance. A 9-F, 45 cm vascular sheath (Cook Medical) was placed over a 0.035-inch guidewire into the right internal jugular vein using the Seldinger technique. Subsequently, a 7-F multipurpose catheter was advanced into the right, middle, or left hepatic vein under fluoroscopic control. Contrast venography was then performed to determine the actual position of the catheter before the biopsy. When performing TJLB, preoperative CT was used to confirm the degree of liver atrophy and the course of major blood vessels, to identify the hepatic vein from which a sample could be safely collected, and to determine the needle direction and distance. Next, venography was performed to confirm the position of the puncture and wedged hepatic venous pressure (WHVP) was measured using a balloon catheter (Terumo, Tokyo, Japan). Using an 18-gauge, 60-cm-long side-cutting automated biopsy device (Quick-Core needle liver access kit; Cook Medical), the needle was positioned in the same direction, and one or several passes were performed to obtain an adequate sample. Fluoroscopy and electrocardiogram monitoring were performed throughout the procedure in both groups.

After the biopsy procedure, a contrast medium was injected through the catheter to rule out capsular perforation. Complications during the procedure were recorded, and the patients’ clinical records were reviewed for any delayed complications.

Statistical analysis

Continuous variables were reported as mean ± standard deviation (SD), whereas categorical data were expressed as frequencies. Student’s t-test was used to compare the continuous variables, whereas Pearson’s chi-squared test or Fisher’s exact test was used to compare the categorical variables between the two groups. P < 0.05 was considered statistically significant.

Data were analyzed using Easy R (EZR) version 1.42 (Saitama Medical Center, Jichi Medical University, Saitama, Japan) [7].

## Results

The laboratory results of participants requiring TJLB were as follows: aspartate transaminase (AST), 270.29 ± 494.46 IU/L; alanine transaminase (ALT), 267.89 ± 466.37 IU/L; total bilirubin, 3.64 ± 5.86 mg/dL; direct bilirubin, 2.84 ± 4.79 mg/dL; alkaline phosphatase (ALP), 818.22 ± 10.49 IU/L; lactate dehydrogenase (LDH), 347.04 ± 852.14 IU/L; γ-glutamyl transpeptidase (γ-GTP), 347.04 ± 399.36 IU/L; platelet count, 13.85 ± 10.19 × 104/μL; and prothrombin time activity, 75.64% ± 21.64%. Ascites were observed in six patients (Table 1).

**Table 1:** Characteristics of patients with hematological diseases and liver dysfunction who underwent transjugular liver biopsy AIHA: autoimmune hemolytic anemia, ALL: acute lymphocytic leukemia, APL: acute promyelocytic leukemia, CMMoL: chronic myelomonocytic leukemia, ITP: idiopathic thrombocytopenic purpura, ML: malignant lymphoma, MM: multiple myeloma, AST: aspartate transaminase, IU/L: international units per liter ALT: alanine transaminase, ALP: alkaline phosphatase, LDH: lactate dehydrogenase, γ-GTP: γ-glutamyl transpeptidase, Plt: platelet count

Categories	Mean ± SD
Age, years	60.07 ± 17.11
Sex (male: female)	12:15
Etiology (AIHA/ALL/Amyloidosis/APL/CMMoL/ITP/ML/MM)	1/1/7/1/1/3/12/1
Prothrombin time, %	75.64 ± 21.64
Total bilirubin, mg/dL	3.64 ± 5.86
Direct bilirubin, mg/dL	2.84 ± 4.79
AST, IU/L	270.29 ± 494.46
ALT, IU/L	267.89 ± 466.37
ALP, IU/L	818.22 ± 610.49
LDH, IU/L	347.04 ± 852.14
γ-GTP, IU/L	347.04 ± 399.36
Plt, ×104/μL	13.85 ± 10.19

The mean age of patients with malignant lymphoma was 59.58 ± 15.07 years; the male-to-female ratio was 5:7. The mean levels of interleukin-2 (IL-2) receptor, AST, ALT, total bilirubin, direct bilirubin, ALP, LDH, γ-GTP; platelet count; and prothrombin time activity were 4023.75 ± 2690.03 U/mL, 205.16 ± 324.33 IU/L, 170.58 ± 194.65 IU/L, 1.66 ± 1.11 mg/dL, 1.08 ± 0.92 mg/dL, 782.16 ± 518.55 IU/L, 964.16 ± 1189.79 IU/L, 376.81 ± 393.69 IU/L; 14.99 ± 11.71 × 104/μL; and 70.5% ± 21.81%, respectively. The levels of IL-2 receptor and LDH were elevated (Table 2). The etiology of liver dysfunction in patients with malignant lymphoma included liver infiltration by the underlying disease (four cases), hepatitis B reactivation (one case), hepatitis E complication (one case), and drug-induced liver injury (six cases). 

**Table 2:** Characteristics of patients with malignant lymphoma and liver dysfunction who underwent transjugular liver biopsy IL: interleukin, AST: aspartate transaminase, IU/L: international units per liter, ALT: alanine transaminase, ALP: alkaline phosphatase, LDH: lactate dehydrogenase, γ-GTP: γ-glutamyl transpeptidase, Plt: platelet count

Categories	Mean ± SD
Age, years	59.58 ± 15.07
Sex (male: female)	5:7
IL-2 receptor, U/mL	4023.75 ± 2690.03
Prothrombin time, %	70.51 ± 21.81
Total bilirubin, mg/dL	1.66 ± 1.11
Direct bilirubin, mg/dL	1.08 ± 0.92
AST, IU/L	205.16 ± 324.33
ALT, IU/L	170.58 ± 194.65
ALP, IU/L	782.16 ± 518.55
LDH, IU/L	964.16 ± 1189.79
γ-GTP, IU/L	376.81 ± 393.69
Plt, ×104/μL	14.99 ± 11.71

The mean age of patients with suspected amyloidosis was 59.28 ± 14.67 years; the male-to-female ratio was 4:3. The mean wedge hepatic venous pressure (WHVP) was high (35.16 ± 7.54 cmH2O). The laboratory test results were as follows: AST, 77.57 ± 51.63 IU/L; ALT, 62.57 ± 47.17 IU/L; total bilirubin, 3.53 ± 7.12 mg/dL; direct bilirubin, 3.12 ± 6.82 mg/dL; ALP, 843.01 ± 701.56 IU/L; LDH, 320.00 ± 176.26 IU/L; γ-GTP, 388.00 ± 460.77 IU/L; platelet count, 18.64 ± 8.48 × 104/μL; and prothrombin time activity, 83.44% ± 20.31%. Additionally, hepatosplenomegaly was indicated, WHVP was found to be high, and jaundice was noted (Table 3). Two patients had findings indicative of nonalcoholic steatohepatitis (NASH). 

**Table 3:** Characteristics of patients with suspected amyloidosis and liver dysfunction who underwent transjugular liver biopsy. WHVP: wedge hepatic venous pressure, AST: aspartate transaminase, IU/L: international units per liter, ALT: alanine transaminase, ALP: alkaline phosphatase, LDH: lactate dehydrogenase, γ-GTP: γ-glutamyl transpeptidase, Plt means platelet count.

Categories	Mean ± SD
Age, years	59.28 ± 14.67
Sex (male: female)	4:3
WHVP, cmH_2_O	35.16 ± 7.54
Prothrombin time, %	83.44 ± 20.31
Total bilirubin, mg/dL	3.53 ± 7.12
Direct bilirubin, mg/dL	3.12 ± 6.82
AST, IU/L	77.57 ± 51.63
ALT, IU/L	62.57 ± 47.17
ALP, IU/L	843.01 ± 701.56
LDH, IU/L	320.00 ± 176.26
γ-GTP, IU/L	388.00 ± 460.77
Plt, ×104/μL	18.64 ± 8.48

The mean age of patients with ITP was 75.00 ± 18.25 years; all three patients were female. The results of laboratory investigations were as follows:; ferritin, 431.50 ± 128.27 ng/mL; AST, 1034.33 ± 681.23 IU/L; ALT, 1071.33 ± 654.14 IU/L; total bilirubin, 5.34 ± 4.35 mg/dL; direct bilirubin, 3.94 ± 3.43 mg/dL; ALP, 662.00 ± 148.84 IU/L; LDH, 484.33 ± 250.04 IU/L; γ-GTP, 156.66 ± 88.56 IU/L; platelet count, 5.80 ± 2.45 × 104/μL; and prothrombin time activity, 80.37% ± 8.88%. The platelet count was reduced, while AST and ALT values were elevated. Jaundice was also observed (Table 4). TJLB results showed findings indicative of autoimmune hepatitis in all patients and steroids were administered. 

**Table 4:** Characteristics of patients with idiopathic thrombocytopenic purpura and liver dysfunction who underwent transjugular liver biopsy AST: aspartate transaminase, IU/L: international units per liter, ALT: alanine transaminase, ALP: alkaline phosphatase, LDH: lactate dehydrogenase, γ-GTP: γ-glutamyl transpeptidase, Plt means platelet count.

Categories	Mean ± SD
Age, years	75.00 ± 18.25
Sex (male: female)	0:3
Ferritin, ng/mL	431.50 ± 128.27
Prothrombin time, %	80.37 ± 8.88
Total bilirubin, mg/dL	5.34 ± 4.35
Direct bilirubin, mg/dL	3.94 ± 3.43
AST, IU/L	1034.33 ± 681.23
ALT, IU/L	1071.33 ± 654.14
ALP, IU/L	662.00 ± 148.84
LDH, IU/L	484.33 ± 250.04
γ-GTP, IU/L	156.66 ± 88.56
Plt, ×104/μL	5.80 ± 2.45

The patient with AIHA was a 79-year-old woman; TJLB was indicated due to thrombocytopenia (platelet count, 22,000/μL). The clinical data were as follows: ferritin, 654.7 ng/mL; AST, 133 IU/L; ALT, 222 IU/L; total bilirubin, 1.45 mg/dL; direct bilirubin, 9.91 mg/dL; ALP, 324 IU/L; LDH, 151 IU/L; γ-GTP levels, 102 IU/L; and prothrombin time activity, 94%. TJLB revealed NASH secondary to drug-induced liver injury.

The patient with ALL was a 34-year-old man. TJLB was indicated due to marked jaundice, with a total bilirubin level of 16.34 mg/dL and a direct bilirubin level of 10.71 mg/dL. The clinical data were as follows: ferritin, 412.6 ng/mL; AST, 20 IU/L; ALT, 85 IU/L; ALP, 1219 IU/L; LDH, 430 IU/L; γ-GTP levels, 1244 IU/L; platelet count, 10.1 × 104/μL; and prothrombin time activity, 53%. Drug-induced cholestatic liver disease was diagnosed using TJLB.

The patient with APL was a 23-year-old woman. The clinical data were as follows: ferritin, 3532.4 ng/mL; AST, 290 IU/L; ALT, 693 IU/L; total bilirubin, 0.61 mg/dL; direct bilirubin, 0.18 mg/dL; ALP, 396 IU/L; LDH, 312 IU/L; γ-GTP levels, 112 IU/L; platelet count, 20.6 × 104/μL; and prothrombin time activity, 105.5%. Drug-induced liver injury was diagnosed using TJLB.

The patient with CMMoL was a 68-year-old man with marked ascites; TJLB was therefore selected. The clinical data were as follows: ferritin, 3532.4 ng/mL; AST, 32 IU/L; ALT, 48 IU/L; total bilirubin, 0.63 mg/dL; direct bilirubin, 0.32 mg/dL; ALP, 2509 IU/L; LDH, 200 IU/L; γ-GTP levels, 176 IU/L; platelet count, 11.8 × 104/μL; and prothrombin time activity, 73%. Liver infiltration by the underlying disease was diagnosed using TJLB.

The patient with multiple myeloma was a 63-year-old man. His platelet count and prothrombin time activity were 1.54 × 104/μL and 35.4%, respectively, indicating marked thrombocytopenia and reduced coagulation ability; thus, TJLB was selected. The clinical data were as follows: ferritin, 1586.2 ng/mL; AST, 715 IU/L; ALT, 486 IU/L; total bilirubin, 18.57 mg/dL; direct bilirubin, 15.08 mg/dL; ALP, 371 IU/L; LDH, 284 IU/L; and γ-GTP levels, 78 IU/L. Liver dysfunction due to disseminated intravascular coagulation was diagnosed using TJLB.

The treatment regimen was modified in patients with liver infiltration by the underlying disease, and the medication regimen was changed in patients with drug-induced liver injury; no complications due to TJLB were observed.

## Discussion

Liver biopsies have been attempted through various techniques, including blind PLB, ultrasound- and CT-guided PLB, and open PLB [8-10]. TJLB is recognized as an alternative biopsy technique to obtain tissue samples [11-15] that can be used in patients with contraindications for PLB, such as those with ascites or a tendency for bleeding. In 1964, Dotter [16] reported the use of TJLB in dogs. In 1967, Hanafee et al. [17] reported the use of transjugular cholangiography. By 1970, Weiner et al. [18] reported the first clinical application of TJLB. Since the report of Rösch et al. [2] on the clinical use of their technique in 1973, there have been numerous reports regarding the procedure. TJLB entails using a biopsy needle guided by a catheter placed in one of the hepatic veins to pierce the liver parenchyma. However, the usefulness of TJLB in cases of hematological diseases complicated by liver disorder has not been sufficiently investigated.

In this study, 27 patients (mean age, 60.07 years; 15 men, 12 women) requiring tissue diagnoses via TJLB after developing liver disorders while undergoing treatment for hematological diseases were enrolled. One patient with autoimmune hemolytic anemia was diagnosed with drug-induced liver injury; two patients with amyloidosis, with nonalcoholic steatohepatitis; one patient with acute promyelocytic leukemia, with drug-induced liver injury; one patient with chronic myelomonocytic leukemia, with liver infiltration caused by the underlying disease; three patients with idiopathic thrombocytopenic purpura, with autoimmune hepatitis; four patients with malignant lymphoma, with liver infiltration by the underlying disease; one patient with multiple myeloma, with liver disorder caused by disseminated intravascular coagulation. Moreover, one patient had hepatitis B reactivation, another had hepatitis E complication, and six patients had drug-induced liver injury. The treatment regimen was altered in cases of liver infiltration caused by the underlying disease, and the drug utilized was changed in cases diagnosed with drug-induced liver injury.

Our results confirmed that TJLB is safe, even for patients with hematological diseases and liver dysfunction. Additionally, tissue sampling using TJLB allowed us to establish a definitive diagnosis in all cases and subsequently formulate an appropriate treatment strategy. Furthermore, previous studies reported that it is possible to simultaneously perform TJLB and measure the hepatic venous pressure gradient [3,4,19]. Herein, we investigated whether the presence of portal hypertension was associated with hematological diseases, including amyloidosis with hepatosplenomegaly. Consequently, we observed that WHVP was elevated in patients with splenomegaly. Six patients with hematological diseases developed ascites; nevertheless, TJLB enabled us to perform histologic diagnosis safely and without bleeding in any of the cases.

The main indications for TJLB were the presence of conditions such as thrombocytopenia, coagulation disorders, ascites, and jaundice. While jaundice itself is not a contraindication for PLB, there is a risk of complications (e.g., biliary peritonitis) in the presence of occlusive jaundice-that is, in cases in which the total bilirubin level exceeds 3 mg/dL (5 mg/dL in this study). However, this risk is extremely rare, and patients with obstructive jaundice are usually decompressed with ERCP is feasible or PTC. We thus determined that TJLB was the preferable option in such cases. Although the ITP cases in this study had a low platelet count, these patients exhibited no bleeding and were diagnosed with autoimmune hepatitis as a complication; consequently, steroids were administered. Jaundice was observed in patients with malignant lymphoma, ITP, and ALL.

While there are no specific contraindications for TJLB, cases with thromboses or other occlusions of the jugular vein, which prevent the catheter from being inserted into the vein, and cases with allergic sensitivity to the contrast medium are generally considered inappropriate for the procedure. Further, TJLB is generally not recommended in cases with lesions in the local region [10].

According to a previous report, tissue samples obtained through TJLB are fragmented, and diagnosis of fragmented tissues is more difficult compared to that of intact tissue samples [20]. However, we previously reported techniques to improve the diagnostic ability of TJLB [14,15], and histological diagnosis was possible in all cases. In addition, TJLB was also found to be useful in a case of lymphoma and advanced thrombocytopenia [21]; thus, even in various cases demonstrating a high risk of bleeding, the use of TJLB allows surgeons to obtain data that would otherwise be difficult to acquire using other procedures. These data are beneficial for diagnosis [22].

Although no adverse events were observed in the present study, it was previously reported that complications associated with TJLB occur at a frequency of 1.3%-20% [1,4,10,23]. Mild complications that have been reported include fever, arrhythmia, and cervical hematoma, while severe complications include pneumothorax, cervical pseudoaneurysm, hemobilia, and intra-abdominal bleeding [24]. Therefore, TJLB is indicated for patients with a high risk of bleeding. It was reported that the frequencies of severe bleeding and death associated with TJLB are 0.01% and 0.02%, respectively; these are lower than the frequencies associated with PLB, which range from 0.16-0.32% [3]. Nevertheless, a study reported two cases of intra-abdominal bleeding in a cohort of 341 patients (0.59%), indicating that caution is required [25]. No severe complications occurred in our study, and TJLB was performed safely in all cases.

Our study has some limitations. First, it included patients with specific hematological diseases and liver dysfunction. Second, this was a single-center retrospective study with a small sample size. Third, it was difficult to evaluate the quality of TJLB in all liver disease cases. Further multicenter studies are warranted to validate our results and further our understanding of the clinical value of TJLB in patients with hematologic diseases and liver dysfunction.

## Conclusions

The etiology of liver disorders in patients with hematological diseases is variable. Percutaneous liver biopsy is contraindicated in these patients due to bleeding risk Transjugular liver biopsy (TJLB) is an alternative method to obtain specimens for histological evaluation and confirm disease etiology. TJLB is a safe option for patients with hematological disease who require a liver biopsy to evaluate the cause of liver dysfunction. This modality allows for diagnostic confirmation and subsequent modification of treatment protocols. Causes of liver disorders in patients with hematological diseases vary greatly. Therefore, TJLB is useful to determine an appropriate therapeutic strategy based on the underlying etiology of liver dysfunction in patients with hematological diseases.
